# Compared the Effectiveness of Intraperitoneal Bupivacaine with Lung Recruitment Maneuver Versus Normal Saline with Lung Recruitment Maneuver in Reducing Shoulder Pain After Laparoscopic Surgery: A Double-Blind Randomized Controlled Trial

**DOI:** 10.5812/aapm-148198

**Published:** 2024-07-14

**Authors:** Javad Shahinfar, Hossein Zeraati, Mahdiyeh Dartoomi, Hosnieh Raoufian

**Affiliations:** 1 Department of Anesthesiology, Emam Ali Hospital, North Khorasan University of Medical Sciences, Bojnurd, Iran; 2 Department of Operating Room, School of Nursing, Torbat Heydariyeh University of Medical Sciences, Torbat Heydariyeh, Iran

**Keywords:** Bupivacaine, Normal Saline, Lung Recruitment Maneuver, Shoulder Pain, Intraperitoneal, Laparoscopy

## Abstract

**Background:**

Postoperative shoulder pain is one of the most common complications following laparoscopic surgery. Various interventions have been proposed to control this pain.

**Objectives:**

The main objective of this comparative study was to determine the effects of intraperitoneal bupivacaine and normal saline infusion, in combination with lung recruitment maneuver (LRM), on shoulder pain following laparoscopic surgery.

**Methods:**

The present randomized controlled trial was conducted on 105 candidates for laparoscopic cholecystectomy referred to Imam Ali Hospital in Bojnurd, Iran, from November 2021 to June 2022. The patients were assigned to three groups using block randomization: BR (receiving 50 cc of 0.25% diluted intraperitoneal bupivacaine + LRM), NR (receiving 50 cc of intraperitoneal normal saline + LRM), and N (receiving 50 cc of intraperitoneal normal saline). Postoperative shoulder pain and surgical incision site pain were assessed using the Visual Analogue Scale (VAS) at recovery intervals of 4, 12, 24, and 48 hours after surgery. Additionally, the prevalence of nausea and vomiting, the first time of need for sedation, and the incidence of sedation overdose in the first 24 hours after surgery were investigated. The data were analyzed using one-way analyzed by one-way analysis of variance (ANOVA) with SPSS software at a significance level of 0.05.

**Results:**

The findings showed no significant differences in demographic variables between the three groups. The range and mean score of shoulder pain based on VAS was 0 - 1 (0.3) in the BR group, 0 - 2 (1.4) in the NR group, and 1 - 3 (2.1) in the N group at 4 hours after surgery. The mean score of shoulder pain intensity in the BR group was lower compared to the NR and N groups during recovery time intervals at 4, 12, and 24 hours after surgery. This difference between groups was significant. There was also a statistically significant difference in the mean score of surgical incision site pain intensity and the first time of need for sedation between the three groups. The occurrence of side effects was not significant between the groups. Itching, bradycardia, and hypotension were not observed in any of the groups.

**Conclusions:**

The findings of this study showed that bupivacaine, along with LRM, is a safe method effective in relieving postoperative shoulder pain. It prolonged the first time of need for sedation and significantly reduced the incidence of shoulder pain.

## 1. Background

Gallstones are common medical conditions of the digestive system (1). Following cystic duct obstruction by gallstones, some patients experience side effects such as acute and chronic cholecystitis, necessitating cholecystectomy. Currently, the gold standard treatment for gallstones is laparoscopic cholecystectomy (2).

Despite this gold standard, many patients complain of shoulder pain (3). The prevalence of shoulder pain following laparoscopy is estimated at 35 to 80%, which may even be worse than the surgical incision site pain (4). Surgical incision site pain and shoulder pain are the main complaints of patients after laparoscopic cholecystectomy and the primary reasons for prolonged hospitalization (4, 5). Incorrect control of postoperative pain can lead to other complications such as hypertension, respiratory problems, and even cardiovascular accidents (6). Therefore, controlling shoulder pain in such patients is of particular importance.

One method of pain control is the use of intravenous analgesics (7), topical drugs, or local anesthetic drugs such as bupivacaine. The local anesthetic bupivacaine provides the benefits of intravenous analgesia without systemic side effects (6). Studies have shown that bupivacaine produces an average of six hours of analgesia (8).

Considering the high use of CO_2_ gas for pneumoperitoneum in laparoscopic surgery and the diaphragm stimulation by the remaining gas at the end of the operation, another method to control shoulder pain is intraperitoneal normal saline infusion. Filling the abdomen with warm normal saline increases CO_2_ released from storage areas in the peritoneal space (9). Another way to reduce shoulder pain is residual CO_2_ evacuation through a lung recruitment maneuver (LRM) at the end of surgery (10).

## 2. Objectives

Considering the importance of reducing shoulder pain in such patients and investigating the effect of safely combined interventions for the most effective method of controlling shoulder pain, the main objective of this comparative study was to determine the effects of intraperitoneal bupivacaine and normal saline infusion plus LRM on shoulder pain following laparoscopic surgery.

## 3. Methods

### 3.1. Study Design and Setting

The current randomized controlled trial was conducted on 105 candidates (aged 20 - 60 years) for laparoscopic cholecystectomy referred to Imam Ali Hospital in Bojnurd (Iran) from November 2021 to June 2022, who met the study inclusion criteria.

Inclusion criteria were: Age between 20 and 60 years, American Society of Anesthesiologists (ASA) Class I and II, no history of heart, respiratory, or kidney diseases, no pregnancy, no history of drug addiction, no history of diseases with chronic pain, no history of hypersensitivity to anesthetic drugs, no history of illness and surgery in the shoulder and chest area, and no history of abdominal surgery, neurological, or mental illness.

Exclusion criteria were: Conversion from laparoscopy to laparotomy and the incidence of any clinical condition preventing the implementation of LRM.

### 3.2. Anesthesia Induction and Maintenance

After the patients entered the operating room, an 18G intravenous cannula was inserted, and Ringer's solution (2 mg/kg) was administered. All procedures for the induction and maintenance of anesthesia were performed uniformly across the three groups. Initially, 0.05 mg/kg of midazolam, 2 μg/kg of fentanyl, and 10 mg of metoclopramide were given as pre-anesthetic drugs. Anesthesia was induced with 2 mg/kg of propofol, and 0.5 mg/kg of atracurium besylate was used as a neuromuscular relaxant. Following preoxygenation and the insertion of a cuffed endotracheal tube, anesthesia maintenance was continued with 1.2 minimum Alveolar concentration (MAC) of inhaled isoflurane along with O_2_ and N_2_O. Minute ventilation was set to maintain ETCO_2_ between 35 and 45 mm Hg. No other analgesics were used during the procedure. At the end of the surgery, the relaxation effects were reversed with neostigmine and atropine.

### 3.3. Surgical Technique

CO_2_ was used as the insufflation gas during laparoscopic surgery. Mean intra-abdominal pressure was maintained between 12 and 15 mmHg (11). The patients were positioned in a 30° reverse Trendelenburg position with a slight lateral tilt to the left. The laparoscopic procedure was performed by an experienced surgeon using standard techniques. At the end of the operation, the pneumoperitoneum was carefully removed through manual compression.

### 3.4. Sample Size and Randomization

The sample size was estimated to be 35 participants per group based on a pilot study and using the formula for "comparing the average of two populations" with a 95% confidence interval and 80% test power. Samples were collected using a convenience sampling method and then allocated to three intervention groups through web-based block randomization. After determining the random sequence in all blocks, cards labeled A (first group), B (second group), and C (third group) were prepared for allocation. These cards were numbered from 1 to 105 by a person outside the research team and placed in non-transparent sealed envelopes.

### 3.5. Intervention Process

The research units were randomly divided into three groups: BR (receiving 50 cc of 0.25% diluted intraperitoneal bupivacaine with distilled water + LRM), NR (receiving 50 cc of intraperitoneal normal saline + LRM), and N (receiving 50 cc of intraperitoneal normal saline). The patients in the BR group received 50 cc of 0.25% diluted bupivacaine, while the NR and N groups received 50 cc of normal saline solution at body temperature. At the end of the surgery, these solutions were injected intraperitoneally under the direct view of the surgeon. Additionally, at the end of the surgery, an anesthesiologist performed a LRM in the NR and BR groups by giving five high-volume breaths to create an airway pressure of 40 cmH_2_O, maintaining the pressure for 5 seconds in the supine position. During this maneuver, the trocar tube valve was fully open to allow CO_2_ to escape. The patients were then placed in a flat position, and the abdominal incisions were closed.

In all patients, the hemodynamic status was evaluated by an anesthesiologist at the end of the operation. If the LRM was deemed not applicable due to the patient's hemodynamic status, the patient would be excluded from the study.

### 3.6. Study Outcomes

The primary outcome of this study was the investigation of shoulder pain along with surgical incision site pain. The secondary outcomes were the prevalence of nausea and vomiting, the first need for sedation, and the occurrence of sedation overdose in the groups. The intensity of postoperative shoulder pain and surgical incision site pain was determined by a Visual Analog Scale (VAS) at recovery intervals of 4, 12, and 24 hours after surgery. The prevalence of nausea and vomiting, the first need for analgesia, and the need for increased analgesic doses in the first 24 hours after surgery were also investigated.

Postoperative pain management was performed uniformly across all groups according to the surgeon's protocol. This included 25 mg of intravenous meperidine in the post-anesthesia care unit (PACU) and 200 mg of oral ibuprofen administered three times daily. Additionally, at the request of the patient, 30 mg of pethidine was provided as a pain reliever in the inpatient department. The time at which the patient requested pain relief was recorded as the first need for sedation.

### 3.7. Blinding Process

To ensure the concealment of random allocation, a sealed opaque envelope was opened at the time of the patient's visit, determining the assignment of each sample to the relevant group. To maintain the double-blind nature of the study, the drug preparation for injection was performed by an anesthesiologist who did not participate in the study. Additionally, both the surgeon and the person performing the outcome assessment were unaware of the group assignments. The patients were also unaware of the type of intervention they received.

### 3.8. Statistical Analysis

The obtained data were evaluated for normality using the Shapiro-Wilk test. Quantitative data were expressed as mean ± standard deviation or median (interquartile range 25% – 75%) and analyzed by one-way analysis of variance (ANOVA). If necessary, a post hoc test was used to determine the level of significance between groups. Univariate analysis of variance was performed to control for possible confounding factors. Descriptive variables were presented as frequency or percentage and were compared via the Chi-square test or Fisher's exact test if necessary.

### 3.9. Ethical Considerations

This study was carried out after obtaining approval from the Ethics Committee of North Khorasan University of Medical Sciences, with the ethics code IR.NKUMS.REC.1399.048, and was registered in the Iranian Registry of Clinical Trials with the code IRCT20190113042346N2.

## 4. Results

**Figure 1.**
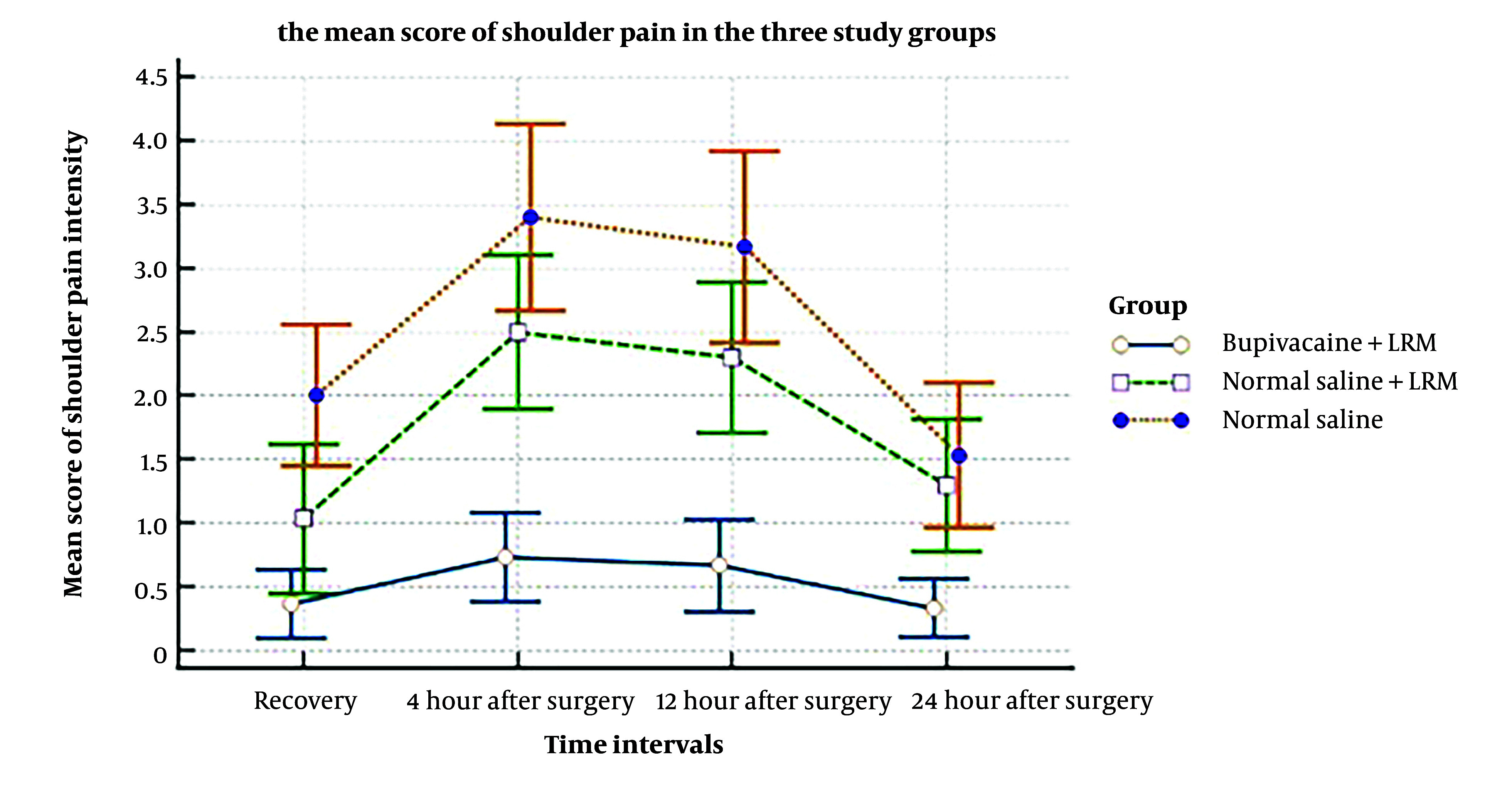
Main score of shoulder pain

In the present study, 4 out of 105 enrolled patients were withdrawn after randomization due to not meeting the inclusion criteria. At the end of the study, 34, 33, and 34 subjects were analyzed in the BR, NR, and N groups, respectively (Figure 1). The mean age of patients in the BR, NR, and N groups was 43.8 ± 9.5, 45.2 ± 10.7, and 46.6 ± 11.4 years, respectively. There was no significant difference in demographic variables and surgery data between the three groups (Table 1). 

**Table 1.** Demographic Variables and Surgical Data in the Three Study Groups ^a^

Variables	Study Groups			Sig
**Bupivacaine + LRM**	**Normal Saline + LRM**	**Normal Saline**
**Age (y)**	43.8 ± 9.5	45.2 ± 10.7	46.6 ± 11.4	0.53
**Weight (kg)**	78.4 ± 13.1	75.9 ± 11.5	76.2 ± 11.9	0.41
**Gender (male-female)**	14.20	15.18	13.21	0.38
**Duration of surgery**	60.7 ± 21.4	64.6 ± 18.3	59.5 ± 19.8	0.28
**Duration of anesthesia**	78.7 ± 26.7	82.3 ± 23.2	79.4 ± 25.5	0.36
**ASA**	30.4	28.7	29.5	0.47

^a^ Values are expressed as mean ± SD.

The findings showed that the mean score of shoulder pain intensity in the BR group was lower than in the NR and N groups at time intervals of 4, 12, 24, and 48 hours after surgery. The range and mean score of shoulder pain based on VAS were 0 - 1 (0.3) in the BR group, 0 - 2 (1.4) in the NR group, and 1 - 3 (2.1) in the N group at 4 hours after surgery. At 24 hours after surgery, the scores were 0 - 1 (0.5) in the BR group, 1 - 4 (2.8) in the NR group, and 2 - 5 (3.7) in the N group. Based on the one-way ANOVA test results, this difference was significant at the evaluation time intervals between the groups.

The mean score of surgical incision site pain intensity was recorded using the VAS tool at specific time intervals in the three groups. There was a statistically significant difference between the three groups at the recovery time intervals of 4, 12, and 24 hours after surgery (Table 2). According to the post hoc test, there was a significant difference in the BR group compared to the NR and N groups during the evaluation intervals.

**Table 2.** The Mean Score of Surgical Incision Site Pain Intensity in the Three Study Groups ^a^

Time Intervals	Study Groups			Sig.
**Bupivacaine + LRM**	**Normal Saline + LRM**	**Normal Saline**
**Recovery **	1.3 ± 0.5	2.6 ± 1.2	2.9 ± 1.4	< 0.05
**4 hours after surgery**	1.6 ± 0.7	3.0 ± 1.1	3.2 ± 1.4	< 0.05
**12 hours after surgery**	1.4 ± 0.6	2.2 ± 0.9	2.4 ± 1.0	< 0.05
**24 hours after surgery**	0.9 ± 0.4	1.6 ± 0.8	1.5 ± 0.9	< 0.05

^a^ Values are expressed as mean ± SD.

The mean time until the first need for analgesia was 202.4 ± 78.3 minutes after surgery in the BR group, 132.8 ± 66.7 minutes in the NR group, and 124.5 ± 59.2 minutes in the N group. According to the post hoc test results, there was a significant difference between the BR group and the other two groups (Table 3). The occurrence of side effects such as nausea and vomiting was not significantly different between the groups. Itching, bradycardia, and hypotension were not observed in any of the groups.

**Table 3.** Postoperative Complications ^a^

Postoperative Complications	Study Groups			Sig.
**Bupivacaine + LRM**	**Normal Saline + LRM**	**Normal Saline**
**First need of analgesia (min)**	202.4 ± 78.3	132.8 ± 66.7	124.5 ± 59.2	< 0.05
**Nausea (frequency)**	2	1	1	> 0.05
**Vomiting (frequency)**	0	0	0	> 0.05

^a^ Values are expressed as mean ± SD.

## 5. Discussion

Researchers today believe that shoulder pain following laparoscopic surgeries is multifactorial. It can be attributed to damage or irritation of the nerves of the diaphragm (due to the production of CO_2_ in the peritoneal cavity) or peritoneal distension, leading to tension and rupture of microvascular structures along with hemorrhage, followed by the release of inflammatory mediators (12).

Several interventions have been used to reduce shoulder pain after laparoscopic cholecystectomy. In the present study, we used three different combined interventions to reduce shoulder pain. The first method was the LRM, which is associated with residual CO_2_ evacuation from the abdomen by increasing the intraperitoneal pressure (12). The second method was the intraperitoneal bupivacaine infusion to reduce visceral pain and peritonitis caused by residual carbonic acid or hemoperitoneum (8). The third approach was the intraperitoneal normal saline infusion; filling the abdomen with warm normal saline increases CO_2_ release from storage areas in the peritoneal space (9).

Several studies have determined the use of LRM in the relief of shoulder pain following laparoscopic surgery. Gungorduk et al. and Garteiz-Martínez et al. investigated the effect of LRM on pain after laparoscopic surgery and found a reduction in postoperative shoulder pain using this maneuver (12, 13). Kiyak et al. determined the effect of semi-fowler positioning in addition to LRM on post-laparoscopic shoulder pain relief among 106 patients aged 18 to 70 years and found that the position of the patients along with maneuvers could intensify its effectiveness in reducing shoulder pain (14). Kumari et al., van Dijk et al., and Davari-Tanha et al. investigated the effect of intraperitoneal normal saline infusion and LRM and reported relief of post-laparoscopic shoulder pain, but LRM was superior to intraperitoneal normal saline infusion for reducing pain in the first 24 hours after laparoscopic surgery (15-17).

We chose bupivacaine for the present study because of its potency and long duration of action. The half-life of bupivacaine has been reported to be between 5 and 16 hours. Since shoulder pain and surgical incision site pain usually peak a few hours after the operation, intraperitoneal injection showed a significant reduction in pain intensity, especially in the first hour. The reason for choosing intraperitoneal injection of bupivacaine is to block visceral afferent signals, potentially alter visceral pain, and generate analgesia (18). The analgesic effect of intraperitoneal bupivacaine has been demonstrated for shoulder pain and surgical incision site pain, in line with our results. Different doses of intraperitoneal bupivacaine have been tested to achieve an effective dose (19, 20). In all studies, a dose of 100 mg of bupivacaine was used, which was effective in pain relief and was associated with a reduction in the use of postoperative analgesics. Other studies used lower doses of bupivacaine, 75 mg, and 50 mg. They found that bupivacaine decreased postoperative shoulder pain for 4 to 8 hours, corresponding to the lower dose used (21, 22). Other studies confirmed our results on the efficacy of bupivacaine for pain relief for 8 hours after laparoscopic surgery (23-25).

Surprisingly, Rademaker et al. could not find any reduction in postoperative pain because the drug was injected in the supine position, which prevented the anesthetization of the phrenic nerve terminus (26). Scheinin et al. (27) and Joris et al. (28) observed no reduction in pain relief in the intervention group, which can be attributed to the low dose of bupivacaine as, in intraperitoneal induction, dose is more important than volume. In the present study, the blood concentration of drugs was not measured, but no systemic side effects were observed, especially since our dose was limited to 50 ccs of 0.25% diluted intraperitoneal bupivacaine. Doses up to 150 mg of bupivacaine are considered relatively safe (29).

Intraperitoneal normal saline caused the abdomen to be filled with warm normal saline, leading to the evacuation of residual CO_2_ from storage areas and effectively washing out the gas. Several studies have examined the use of normal saline in combination with other approaches, but limited studies have used normal saline alone. The results indicate that the combined effect of normal saline with other methods is more effective, and using this method alone will not be as effective in controlling pain. Ryu et al. compared intraperitoneal normal saline (N/S) with and without LRM on post-laparoscopic shoulder pain among 48 patients. Their findings showed that using normal saline alone had a greater effect on postoperative shoulder pain than the combined method of normal saline and lung maneuver, which is inconsistent with our study (30). Other studies suggest using intraperitoneal N/S in combination with other methods for pain control (16, 17).

Due to the increase in the number of laparoscopic interventions and the high incidence of shoulder and abdominal pain after laparoscopic surgery, further interventions are needed to reduce post-laparoscopic pain and provide satisfactory medical care. The research results revealed that all three methods—normal saline + LRM, bupivacaine + LRM, and normal saline alone—were effective in reducing shoulder pain after laparoscopic cholecystectomy, but the combined method of bupivacaine + LRM had a greater effect on pain relief. There were some limitations to our study. First, we used the same dose of bupivacaine and normal saline, while further research is needed to determine the appropriate intraperitoneal dose. Second, our study had a relatively small sample size. Third, we did not evaluate the effect of patient position on the administration of drugs or the injection of drugs before cholecystectomy. Finally, we did not evaluate the extubation and recovery times affected by the study drugs.

Samarah et al. demonstrated that the use of the recruitment maneuver significantly reduced shoulder pain following laparoscopic cholecystectomy. In this study, the intervention group experienced less shoulder pain compared to the control group (31). Similarly, the study by Temtanakitpaisan et al. found comparable results in their research (32). Iqbal et al., in their study, showed that using bupivacaine at the site of laparoscopic surgery not only reduced pain in the surgical area but also significantly decreased shoulder pain after surgery (33).

It is recommended that future studies investigate other local anesthetic drugs, either alone or in combination with the recruitment maneuver, to reduce shoulder pain after laparoscopy. Additionally, the role of shoulder pain reduction in post-surgery sedation and during recovery should be examined in future studies.

The findings of this study showed that bupivacaine along with the LRM is a safe method effective in relieving postoperative shoulder pain. It also prolonged the first time of need for sedation and reduced the incidence of shoulder pain.

## Data Availability

The dataset presented in the study is available on request from the corresponding author during submission or after its publication. The data are not publicly available due to restrictions, e.g., privacy or ethics.

## Clinical Trial Registration Code:

IRCT20190113042346N2.
